# Cone beam computed tomography for detecting residual stones in percutaneous nephrolithotomy, a randomized controlled trial (CAPTURE) protocol

**DOI:** 10.1186/s13063-021-05794-5

**Published:** 2021-11-15

**Authors:** R. A. Kingma, I. J. de Jong, M. J. W. Greuter, S. Roemeling

**Affiliations:** 1 Department of Urology, University Medical Center Groningen, University of Groningen, Groningen, The Netherlands; 2 Department of Radiology, University Medical Center Groningen, University of Groningen, Groningen, The Netherlands

**Keywords:** Cone beam computed tomography, Percutaneous nephrolithotomy, Residual fragments, Urolithiasis, Endo-urology, Hybrid operating room

## Abstract

**Introduction:**

Percutaneous nephrolithotomy (PCNL) is the standard surgical treatment method for large kidney stones. Its aim is to achieve a stone-free status, since any residual fragments (RFs) after PCNL are likely to cause additional morbidity or stone growth. Enhancing intraoperative detectability of RFs could lead to increased stone-free rates and decreased re-intervention rates. Cone beam computed tomography (CBCT) has recently been introduced in urology as a feasible method for intraoperatively imaging RFs. The aim of this trial is to determine the added value of CBCT in percutaneous nephrolithotomy, by measuring differences in stone-related morbidity for patients with procedures in which a CBCT is used versus patients with procedures without the use of CBCT.

**Methods:**

The CAPTURE trial is an investigator-initiated single-center, randomized controlled trial (RCT) in adult patients who have an indication for percutaneous nephrolithotomy. A contemporary percutaneous nephrolithotomy is performed. Once the surgeon is convinced of a stone-free status by means of fluoroscopy and nephroscopy, randomization allocates patients to either the study group in whom an intraoperative CBCT scan is performed or to the control group in whom no intraoperative CBCT scan is performed. The main endpoint is the stone-free status as assessed four weeks postoperatively by low-dose non-contrast abdominal CT, as a standard follow-up procedure. Secondary endpoints include the number of PCNL procedures required and the number of stone-related events (SREs) registered. The total study population will consist of 320 patients that undergo PCNL and are eligible for randomization for an intraoperative CBCT scan.

**Discussion:**

We deem a randomized controlled trial to be the most effective and reliable method to assess the efficacy of CBCT in PCNL. Though some bias may occur due to the impossibility of blinding the urologist at randomization, we estimate that the pragmatic nature of the study, standardized circumstances, and follow-up methods with pre-defined outcome measures will result in a high level of evidence.

**Trial registration:**

Netherlands Trial Register (NTR) NL8168, ABR NL70728.042.19. Registered on 15 October 2019. Prospectively registered.

## Administrative information

Note: the numbers in curly brackets in this protocol refer to SPIRIT checklist item numbers. The order of the items has been modified to group similar items (see http://www.equator-network.org/reporting-guidelines/spirit-2013-statement-defining-standard-protocol-items-for-clinical-trials/).

**Table:** 

Title {1}	Cone beam computed tomography for detecting residual stones in percutaneous nephrolithotomy, a randomized controlled trial (CAPTURE) protocol
**Trial registration {2a and 2b}.**	Netherlands Trial Register, NTR (trialregister.nl): NL8168, ABR NL70728.042.19. Registered on 15 October 2019. Prospectively registered.
**Protocol version {3}**	Version 4; 16.03.2021
**Funding {4}**	None to declare
**Author details {5a}**	^1^Department of Urology, University Medical Center Groningen, University of Groningen, the Netherlands. ^2^Department of Radiology, University Medical Center Groningen, University of Groningen, the Netherlands.
**Name and contact information for the trial sponsor {5b}**	University Medical Center Groningen , University of Groningen, PO Box 30.001 9700 RB Groningen, the Netherlands, researchsupport@umcg.nl
**Role of sponsor {5c}**	The sponsor played no part in study design and will play no part in the collection, management, analysis and interpretation of data, writing of the report and the decision to submit the report for publication.

## Introduction

### Background and rationale {6a}

Urolithiasis is one of the most common urological diseases and its prevalence has increased over the past decades [1, 2]. In the USA, approximately 1 in 11 people are affected by kidney stones [3]. Emergency department visit rates for urolithiasis have increased by over 90% in the period from 1992 to 2009 [4]. This increasing burden of disease aggravates the need for improving treatment methods for urolithiasis.

The main surgical treatment modality for large (> 2 cm in diameter) or complex kidney stones is percutaneous nephrolithotomy (PCNL) [5]. In PCNL, the kidney is punctured from the flank and dilation of this tract allows for percutaneous access to the kidney. Through this direct rigid access method, stone fragments can be fragmented with powerful fragmentation techniques and stone fragments can be evacuated.

The primary goal of PCNL is a complete removal of stone material, since any left residual fragments (RFs) may cause recurrence of initial symptoms [6, 7].

The practice of PCNL has changed and improved significantly over the years, along with the experience of surgeon s[8, 9]. Access tract diameters have decreased, resulting in less kidney trauma and bleeding. Furthermore, stone fragmentation methods, grasping devices, and baskets have been improved.

In terms of diagnostics of urolithiasis, a shift towards the use of computed tomography (CT) is seen, since it is the most reliable diagnostic tool for imaging urinary stones**.** Radiation exposure has been drastically lowered by developing low-dose non-contrast helical CT protocols. A downside of this imaging modality, however, is that standard helical CT scanners cannot be used intraoperatively.

During a standard PCNL, the surgeon has X-ray fluoroscopy, ultrasonography, and nephroscopy at his or her disposal. However, both fluoroscopy and ultrasonography have a low sensitivity for small or soft stone fragments [10] and full inspection of the renal collecting system by means of nephroscopy can be difficult due to the sinuous nature of the renal anatomy or due to vision impairment caused by bleeding. This leads to a false assumption of a stone-free status in approximately 20% of PCNL cases [11]. Increasing the intraoperative detectability of residual stones could be a major advancement towards increasing stone-free rates and could thereby significantly reduce stone-related morbidity.

As CT is the preferred imaging modality for urinary stones, it would be of great value to enable its use intraoperatively. With the emergence of hybrid operating rooms, cone beam computed tomography (CBCT) has made its entry into several fields of medicine. CBCT scanners do not require the patient or radiation source to translate, as a result of their cone-shaped radiation beam as compared to the conventional fan-shaped beam. The scanner makes a rotation of approximately 200 degrees, and from the detected attenuation profiles image reconstructions can be made that can be viewed cross-sectionally and three-dimensionally.

CBCT has recently been introduced in urology. It has been described in PCNL for the use of needle guidance for gaining percutaneous acces s[12, 13] or for the detection of residual stones [14]. A recently published paper describing a study conducted in our center demonstrated the feasibility of CBCT for the detection of residual stones in PCN L[15]. In this study, we included 19 PCNL procedures with an intraoperative CBCT in order to be able to detect RFs. In 9 out of 19 PCNL procedures, additional stone extraction occurred after making the CBCT scan. Stone-free rates in our study were higher (63% versus 47%) on a 4-week follow-up CT as compared to the CBCT scan.

The evidence that is available to date shows that CBCT in PCNL could increase stone-free rates, while imposing an acceptable burden on patients and the health care system, in terms of radiation dose, additional surgery time, and costs [12–15]. However, several limitations apply to the studies that have already been conducted.

First of all, sample sizes of these studies were relatively small and contained a diverse case-mix, thereby weakening the strength of the results. As of our knowledge, no study has yet evaluated the intraoperative use of CBCT in PCNL with a sample size above 20 patients. Second, investigator bias was not avoided, since the surgeon knew beforehand that a CBCT scan would be made in every case. Thereby, the outcomes of the CBCT scans and additional extraction of stone material could have been influenced.

One could argue that this bias could be bypassed by comparing the (long-term) outcomes of patients in which a CBCT scan was made to aid in residual stone detection with a retrospective cohort without the use of the CBCT scanner. However, the PCNL technique and follow-up methods are rapidly evolving, therefore making it impossible to achieve a comparable control cohort. New stone fragmentation methods have entered the market, and follow-up protocols have altered in both intervals and imaging methods for assessment of the stone-free status.

Therefore, we deem the only effective method to analyze the efficacy of CBCT in PCNL to be a prospective randomized study.

### Objectives {7}

The primary objective of this trial is to determine the added value of CBCT in PCNL, by measuring differences in stone-free rates and stone-related morbidity in patients in whom a CBCT scanner is used versus patients in whom this is not the case.

A secondary objective is to determine whether the use of CBCT in PCNL leads to a significant decrease in the total number of procedures needed per stone case as compared to procedures in which no CBCT scanner is used. Furthermore, we will observe the number of stone-related events (SREs) for patients in both groups, in order to assess the benefits for patients from the use of the CBCT scanner during their intervention.

SREs in this study are defined as follows: recurrence of symptoms, urinary tract infections, stone-related hospital admissions, emergency visits, and re-interventions.

### Trial design {8}

This study is a non-blinded, single-center randomized controlled trial comparing the potential benefits of an intraoperative CBCT scan during PCNL against a standard PCNL without the use of CBCT.

## Methods: participants, interventions, and outcomes

### Study setting {9}

We are currently recruiting consecutive patients in the University Medical Center Groningen (UMCG) that will undergo percutaneous nephrolithotomy (PCNL) for kidney stones.

### Eligibility criteria {10}

In order to be eligible to participate in this study, a subject must meet all of the following inclusion criteria:

- ≥18 years of age
- Availability of a non-contrast abdominal CT scan with presence of kidney stones
- Consent by urologist and patient that a PCNL is the appropriate method for stone removal
- Able and willing to undergo PCNL and 1 year of follow-up
- Capacity to give informed consent to participate in the trial
- Fluent in Dutch or English language

A potential subject who meets any of the following criteria will be excluded from participation in this study:

- Pregnancy

### Drop-out criteria

Some participants may drop-out during study participation. The drop-out criteria for this study are:

- Not eligible for randomization
- Loss to follow-up
- Withdrawal of participation by participant

### Who will take informed consent? {26a}

Consent is taken by the urologist-researcher at the out-patient department, who is trained in Good Clinical Practice (GCP). This will occur during the out-patient hospital appointment, at the point when a potential participant is scheduled for PCNL.

### Additional consent provisions for collection and use of participant data and biological specimens {26b}

The consent form asks participants for permission to contact them in the future for possible participation in further research projects and for permission to use the data that will be collected in this trial for future studies. Declining either of these options has no effect on participation in the CAPTURE trial.

## Interventions

### Explanation for the choice of comparators {6b}

The comparator is an intraoperative CBCT scan during PCNL. Justification for this is given in the “Background and rationale {6a}” section.

### Intervention description {11a}

#### Trial arms

The trial consists of two arms: an arm with patients receiving an intraoperative CBCT scan and an arm with patients that do not receive an intraoperative CBCT scan. The surgery procedure and preparation will be equal for both arms. At the end of the procedure, at the point where the urologist would normally finish the procedure if he would not have a CBCT scanner at his disposal, the randomization will be performed intraoperatively through an internet connection with the randomization software. Patients will be randomized in a 1:1 allocation to either the “CBCT scan” or “no CBCT scan” arm.

#### CBCT scanner and protocols

The CBCT scanner in the urological hybrid operating room of our center is an ArtisQ Ceiling DynaCT (Siemens Healthineers). A dedicated workstation performs three-dimensional image reconstruction and image postprocessing. The CT protocol that is used has a radiation exposure time of 6 seconds, with a total of 397 frames and a radiation dose of 0.36 μGy/f. Slice thickness is set at 2 mm, with an increment of 1 mm. As for reconstruction parameters, a “Hounsfield Units (HU) Kernel” is used and image characteristics are set to “Normal.”

#### Follow-up procedure

For all patients, either in the CBCT-arm or non-CBCT-arm, a 4-week follow-up visit will be scheduled with a follow-up CT. At this appointment, several data, such as postoperative complications, patient complaints, stone analysis outcomes, and the outcome of the follow-up CT will be collected and stored in RedCAP. Several options for further follow-up are possible, depending on the outcomes after PCNL.

#### End of trial

The end of participation in the trial for each participant is defined as having completed the 12-month follow-up visit. Patients may or may not have consented to further data collection for prolonged observation. The data that will be collected in this way can be used for subsequent follow-up trials.

### Criteria for discontinuing or modifying allocated interventions {11b}

No special criteria for discontinuing or modifying allocated interventions are present. Participants in the CBCT-arm will receive an intraoperative CBCT scan, whereas participants in the comparison group will undergo the PCNL procedure according to standard care, which is without a CBCT scan.

### Strategies to improve adherence to interventions {11c}

None. Since the intervention occurs intraoperatively and directly after randomization, we expect no non-adherence to interventions.

### Relevant concomitant care permitted or prohibited during the trial {11d}

Standard care for participants in both arms will continue throughout the trial. There are no special restrictions or permissions.

### Provisions for post-trial care {30}

None beyond standard care.

### Outcomes {12}

#### Primary outcome measure

The primary outcome measure is the stone-free status on follow-up CT four weeks postoperatively. The follow-up CT will be examined by an independent radiologist who is unaware of treatment allocation. The stone-free rates will be calculated into mean stone-free percentages per arm.

#### Secondary outcome measures

Secondary outcome measures are:

- The number of PCNL procedures required per episode of 12 months starting from the first PCNL procedure.
- Stone-free status at the latest point within an episode of 12 months as assessed by low-dose abdominal CT.
- Number of SREs registered within a period of 12 months.
- Number of re-interventions registered within a period of 12 months.
- Duration until SRE.
- Duration until re-intervention.
- Duration of surgery.
- Perioperative complications.

### Participant time line {13}

**Fig. 1:**
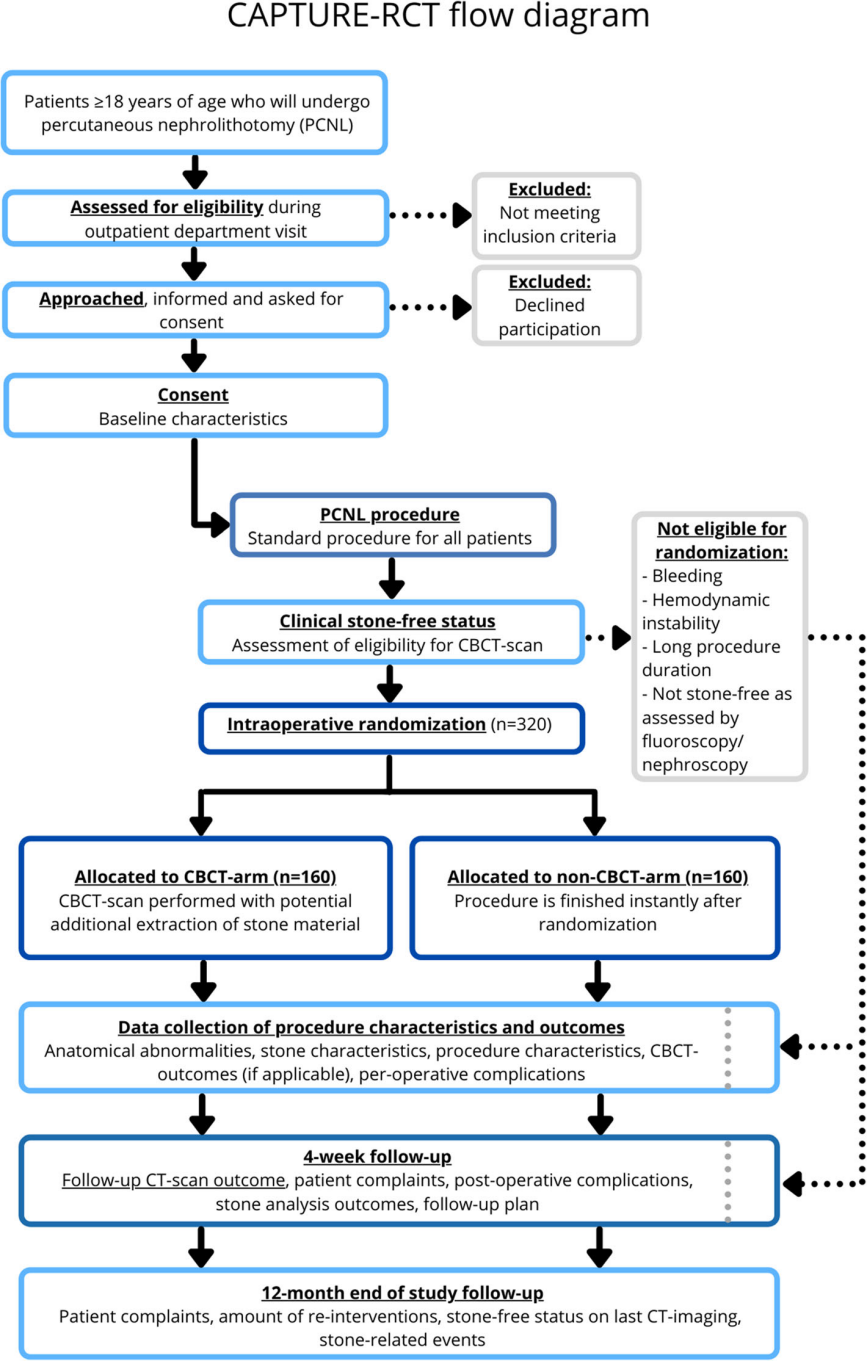
Flow diagram of the study design of the CAPTURE randomized controlled trial

Figure 1 shows a flow diagram of the study design and participant’s time line through the trial.

### Sample size {14}

In our feasibility study, the absolute increase of stone-free rate was 16%, with an increase in stone-free rates from 47 to 63%. However, average one-step stone-free rates in literature are higher. For instance, Atmoko et al. report a one-step stone-free rate of 62.6% for staghorn stone s[16]. The stone-free rates in our pilot study were low due to difficult cases with a small study population. Therefore, we based our sample size calculation on a difference of 60% to 75%, with a difference of 15% as the desired difference to prove. With a power of 80% and alfa 0.05 the estimated sample size per arm is 152 patients, resulting in a total sample size of 304 patients. With a 5% margin for possible unexpected loss of patient data, this results in an intended sample size of 320 patients. On average, 80–100 PCNL procedures are performed in our center each year. Taking into account that in some procedures no randomization can be performed, we estimate our rate of inclusion at 60–70 patients per year.

### Recruitment {15}

All eligible patients will be informed about the study during the appointment in which they are scheduled for PCNL. From this point, they will have a minimum of 2 weeks to decide upon participation or not and to be able to ask any questions about the trial. After 2 weeks, the research nurse will call the screened participants by telephone and answer any further questions they may have. Informed consent forms will then be signed by the participant and a researcher.

## Assignment of interventions: allocation

### Sequence generation {16a}

Randomization will occur intraoperatively. It will be performed with the Research Electronic Data Capture (REDCap) randomization module. REDCap is a secure, web-based software platform designed to support data capture for research studies. REDCap offers a randomization module within the data capture system. A blocked randomization sequence is used with randomly sequenced blocks in varying sizes in a 1:1 allocation. No stratification factors are used in the randomization sequence. In some cases, participants will not be eligible for randomization. This occurs when the procedure must be ended for safety reasons (e.g. bleeding, anesthesiological issues, long procedure duration) or when is known that making a CBCT scan would have no effect, for instance when no clinically stone-free status can be achieved. In order to keep the analyses representative for the whole PCNL population, the procedures in which no randomization could be performed will be recorded as well. In this way, insight is provided into the frequencies and causes of not being able to use the CBCT scanner.

### Concealment mechanism {16b}

The REDCap randomization module ensures concealment of the allocation sequence.

### Implementation {16c}

The randomization sequence is unknown to the research members and can only be accessed by the independent REDCap administrators. A trained nurse will perform randomization during the procedure, by logging in to REDCap and filling out the randomization form for the particular study subject. The nurse will double check the randomization allocation with a colleague and communicate the outcome (CBCT scan or no CBCT scan) with the urologist.

## Assignment of interventions: blinding

### Who will be blinded {17a}

This trial is non-blinded. Blinding of the urologist performing the procedure is impossible, since the urologist needs to perform the CBCT scan intraoperatively and interpret it. Since the additional value of blinding the participants seems small, we deem it unethical to withdraw this information from them and therefore have opted to disclose information about the surgery procedure and randomization to the participants after the procedure.

### Procedure for unblinding if needed {17b}

Not applicable.

## Data collection and management

### Plans for assessment and collection of outcomes {18a}

Data are collected at baseline, at the PCNL procedure, at 4 weeks postoperatively, and at 12 months postoperatively.

### Plans to promote participant retention and complete follow-up {18b}

All kidney stone patients are scheduled to undergo follow-up for at least one year in our center. No additional visits specifically for this trial will be scheduled since data is collected at standard of care follow-up visits. This ensures a low burden for participants and promotes participant retention.

### Data management {19}

All data will be entered and stored by site staff in a REDCap database that is designed specifically for this trial. All participants are given an individual trial number which will be used within the database. Acquired data will be kept for 15 years as is an ethical standard in our center.

### Confidentiality {27}

Data are stored in accordance with GCP and the General Data Protection Regulation (GDPR).

### Plans for collection, laboratory evaluation, and storage of biological specimens for genetic or molecular analysis in this trial/future use {33}

Not applicable.

## Analysis

### Statistical methods for primary and secondary outcomes {20a}

All statistical analyses will be performed using the Statistical Package for the Social Sciences for Windows (SPSS, version 23.0). Descriptive statistics will be presented using mean (± SD) for normally distributed variables and median (IQR) in case variables are not normally distributed. Continuous variables will be compared using *t* tests, whereas for categorical variables, a chi-square test or Fisher’s exact test will be used to compare proportions. A comparison of the primary outcome, the stone-free status on follow-up CT four weeks postoperatively, will be performed with a Chi-square test of the means. A Kaplan-Meier survival analysis will be performed for the survival of SREs and survival of re-interventions per treatment arm, with a log-rank test to assess for statistical significance. For all statistical analyses, an alpha of 0.05 will be used.

### Interim analysis {21b}

When half of the intended study population has been reached, at 160 randomized participants, an interim analysis will be performed. The results of this analysis will determine if the study will be either continued or prematurely terminated, either due to futility or superiority. The difference in stone-free rates will be calculated alongside a 95% confidence interval (CI). The study will be terminated due to futility if the upper limit of this CI is below 5% or terminated due to superiority if the lower limit is above 15%. Interim analysis will be performed by the project leader. The results will be accessed and monitored by the data monitoring committee, after which the outcome (continuation or termination) of the trial will be concluded.

### Methods for additional analyses (e.g., subgroup analyses) {20b}

In order to assess the ideal PCNL characteristics for performing a CBCT-guided PCNL, subgroup analyses will be performed in order to assess the primary and secondary outcome measures for different stratification variables. These stratification variables include the surgery characteristics, stone size, stone type and stone location.

### Methods in analysis to handle protocol non-adherence and any statistical methods to handle missing data {20c}

Analysis will be by intention-to-treat. We have no intention of imputing missing values. We will assess for patterns in possible missing data but expect any missing data to be missing at random, since the follow-up protocols are identical for both study arms.

### Plans to give access to the full protocol, participant level-data and statistical code {31c}

The full protocol, trial data and statistical code may be accessed upon request by contacting the corresponding author.

## Oversight and monitoring

### Composition of the coordinating center and trial steering committee {5d}

The trial steering committee consists of the principal investigator and the project leader. The principal investigator and project leader meet on a weekly basis. The trial is evaluated by a trial steering committee consisting of both research team members and independent researchers in monthly research meetings. The protocol and statistical analysis plan were reviewed by an independent statistician, who is not a definite member of the trial steering committee.

### Composition of data monitoring committee, its role, and reporting structure {21a}

The Data Monitoring Committee will monitor the safety and trial progress independently. The data monitoring committee consists of two trained independent data monitors. The data monitoring committee will perform independent data verification, check randomization procedures and confirm statistical analyses independently. It will report to the Medical Ethics Committee of our center and to the research team.

### Adverse event reporting and harms {22}

The main risks for participants consist of an added radiation exposure and added surgery time if allocated to the interventional group. The added radiation exposure that is expected is calculated to be 1.5–2.0 mSv per CBCT scan. This effective dose corresponds to a minor to intermediate risk category IIb, where the benefit for the participant is aimed at cure or prevention of disease according to the ICRP publication 6 2[17]. Surgery time needed for preparation, performing, and interpreting an intraoperative CBCT scan is around 8 minutes, according to our recently conducted pilot study. If imaged RFs are extracted, the median total extra surgery time needed in that pilot study was 20 min. This added time increases the anesthesia duration and could thereby increase the risk of perioperative complications.

Adverse events (AEs) that may occur can be a result of the risks mentioned above. All AEs will be registered in the REDCap database. Assessment of AEs will be performed and any serious AEs (SAEs) will be reported. The sponsor will report SAEs through the web portal “ToetsingOnline” to the accredited Medical Ethical Committee that approved the protocol, within 7 days of first knowledge for SAEs that result in death or are life-threatening followed by a period of a maximum of 8 days to complete the initial preliminary report. All other SAEs will be reported within a period of maximum 15 days after the sponsor has first knowledge of the SAEs.

### Frequency and plans for auditing trial conduct {23}

This study has been approved by the local Medical Ethics Committee as a low-risk study (Risk Classification 1). The database will be monitored once yearly according to standard ethical practice for a low-risk study clinical trial in our center. Monitoring is performed by a trained monitor from our center who is independent. Plans for communicating important protocol amendments to relevant parties (e.g., trial participants, ethical committees) {25} of our department. The Medical Ethics Committee will receive a yearly update of the status of the trial. Any protocol amendments will be submitted to the Medical Ethics Committee for approval.

### Protocol amendments

No relevant protocol amendments have been implemented yet.

### Ancillary and post-trial care

The care that participants receive does not differ from standard care apart from the randomization with a possible intervention and data collection. No specific measures are undertaken for ancillary or post-trial care.

### Dissemination plans {31a}

We plan to release the study results to referring physicians, participants, future patients, and the general medical community. We seek to reduce the interval between completion of data collection and dissemination of study results to a minimum.

## Discussion

This study will be the first randomized trial evaluating the use of CBCT during PCNL. With a design that standardizes and equalizes circumstances for both arms except for the intervention that is to be evaluated, the imposed burden for participants is kept low while maintaining a high level of evidence.

To date, it is unclear whether the use of CBCT during PCNL can genuinely increase stone-free rates and decrease stone-related morbidity. A positive result of the proposed trial could lead to the adaptation of hybrid operating rooms for PCNL on a larger scale. The results will aid in indicating the circumstances in which performing a “hybrid PCNL” will result in a greater chance of a positive outcome.

Several limitations should be noted for this study. First of all, the unblinded urologist performing the procedure could affect the postoperative outcomes, thereby possibly influencing the secondary outcome measures for this trial. The primary outcome, however, the stone-free status on a 4-week postoperative CT scan, is an objective outcome measure that cannot be influenced in this way.

An ethical dilemma that arises with regard to the design of this study, is that with a relatively large sample size and a 1:1 randomization allocation ratio, a substantial number of participants could be deprived of a potentially superior treatment. Considering possible downsides, e.g., high costs, additional procedure duration, and radiation exposure, a study with a high level of evidence is required in order to conclude whether this technique should be either implemented or rejected.

The interim analysis imposes a limitation for this study, since analyzing the data multiple times increases the chance of occurrence of a Type I error. In this study, no alpha adjustments are made besides setting broad termination boundaries including 95% confidence intervals. This was done in order to maintain a reasonable chance of detecting superiority while maintaining a commonly used alpha of 0.05 at the final analysis.

Another limitation concerns the long duration of the trial caused by a large sample size in a single-center setting. Since PCNL methods, CBCT scanners, and follow-up protocols may vary widely per center, analysis of data from multiple centers would become a challenge. Along with this, not many centers have a dedicated urological hybrid operating room at their disposal that allows for performing a hybrid PCNL in every case, which is required for this study design. In this trial, we expect a high rate of participation and a low rate of exclusion, resulting in a database of nearly every PCNL performed in our center. Therefore, this trial provides a basis of data for a follow-up period of many years to come, since we expect most participants to consent to longitudinal data collection. On that account, we consider this trial to be a valuable investment that is likely to result in a broad range of future research opportunities in the field of kidney stone surgery.

## Trial status

The first participant was recruited in January 2020 and the trial is currently open to recruitment. The current protocol version is version 4, 16 March 2021. Recruitment is expected to complete in 2026.

## Abbreviations

AE: Adverse event
CBCT: Cone beam computed tomography
CT: Computed tomography
GCP: Good Clinical Practice
GDPR: General Data Protection Regulation
HU: Hounsfield Units
NTR: Netherlands Trial Register
PCNL: Percutaneous nephrolithotomy
RCT: Randomized controlled trial
REDCap: Research Electronic Data Capture
RF: Residual fragment
SAE: Serious adverse event
SPSS: Statistical Package for the Social Sciences
SRE: Stone-related event
UMCG: University Medical Center Groningen
